# Growth of Highly-Ordered Metal Nanoparticle Arrays in the Dimpled Pores of an Anodic Aluminum Oxide Template

**DOI:** 10.3390/nano12223929

**Published:** 2022-11-08

**Authors:** Gavin Farmer, James Abraham, Chris Littler, A. J. Syllaios, U. Philipose

**Affiliations:** Department of Physics, University of North Texas, Denton, TX 75077, USA

**Keywords:** Templated, nanoparticle, aluminum oxide

## Abstract

A reliable, scalable, and inexpensive technology for the fabrication of ordered arrays of metal nanoparticles with large areal coverage on various substrates is presented. The nanoparticle arrays were formed on aluminum substrates using a two-step anodization process. By varying the anodization potential, the pore diameter, inter-pore spacing, and pore ordering in the anodic aluminum oxide (AAO) template were tuned. Following a chemical etch, the height of the pores in the AAO membrane were reduced to create a dimpled membrane surface. Periodic arrays of metal nanoparticles were subsequently created by evaporating metal on to the dimpled surface, allowing for individual nanoparticles to form within the dimples by a solid state de-wetting process induced by annealing. The ordered nanoparticle array could then be transferred to a substrate of choice using a polymer lift-off method. Following optimization of the experimental parameters, it was possible to obtain cm${}^{2}$ coverage of metal nanoparticles, like gold and indium, on silicon, quartz and sapphire substrates, with average sizes in the range of 50–90 nm. The de-wetting process was investigated for a specific geometry of the dimpled surface and the results explained for two different film thicknesses. Using a simple model, the experimental results were interpreted and supported by numerical estimations.

## 1. Introduction

Ordered arrays of metal nanoparticles exhibit unique optical and plasmonic properties, making them attractive for applications ranging from energy harvesting to chemical and bio-sensing [1], medical imaging and therapy [2], as well as in magnetic memory arrays. In the past several decades, the phenomenon of localized surface plasmon resonance (LSPR) [3] in such arrays has been studied extensively, specifically with respect to light-matter interactions that provides the framework for conversion of light into electrical signals. Progress in this field allows for a merger between nanoscale photonics and electronics and is essential for the development of next generation opto-electronic devices [4,5]. For noble metal nanoparticles, the LSPR occurs within the visible and IR regime, making them suitable for use in various sensor technologies, since these regions of the spectrum are easily accessed.

The plasmonic properties of the metal nanoparticle arrays are highly sensitive to their spatial characteristics in terms of particle size, inter-particle distance and ordering. Therefore achieving reproducible and reliable control of the experimental parameters that determine the array characteristics is essential. One of the challenges faced in fabricating ordered metal nanoparticle arrays is achieving consistent ordering with uniform particle size over large areas. Typical fabrication methods include electron beam [6], nano-imprint [7], and ion beam lithography [8], but achieving large areal coverage with these methods is time-consuming and expensive [9]. Colloidal lithography and hole-masked colloidal lithography [10] are other techniques which are capable of creating ordered array fabrication over larger surface areas; however they are limited by a lack of control over particle shape and size [11]. Colloidal synthesis of nanoparticles [12] can result in uniformly sized nanoparticles; however their arrangement on a substrate of choice is challenging. Farzinpour et al. [13] have used a mask-based approach that displays control over nanoparticle size, however it does not allow for the fabrication of ordered arrays.

In this work, a template-based method was used to fabricate a nano-dimpled surface that was subsequently used to create an ordered array of uniform sized metal nanoparticles. Template-based synthesis methods have been used to produce different forms such as nanoparticles, nanowires, nanotubes, nanoflakes, and nanosheets. The advantages of using a nano-dimpled aluminium template are the following: (i) the process results in highly ordered distributions, where the dimple size and the evaporated metal film thickness controls the nanoparticle size and spacing; (ii) the relatively inexpensive and reproducible technology can produce large surface area (in the cm${}^{2}$ range) nanoparticle coverage; (iii) the direct transfer of the nanoparticles to a substrate of choice using a polymer-based peel-off method is possible; (iv) the simple technique does not involve the use of any complex tools.

There are published works that demonstrate solid state de-wetting (agglomeration) as a simple and scalable approach for fabrication of ordered arrays of metal nanoparticles [14,15,16,17]. In this technique, the deposited thin metal film, when heated to sufficiently high temperatures (well below the metal’s melting temperature), will agglomerate on the substrate surface to form islands. In the absence of a patterned surface this process, driven by surface energy minimization, will result in randomly distributed islands, since de-wetting mostly occurs at defects and grain boundaries [18]. In order to enable ordering of the nanoparticles, the process was modified to control the de-wetting of the metal film by disrupting the continuity of the deposited film using a surface patterned with an ordered array of pores or dimples [19,20]. It has been shown that when using the AAO template for the de-wetting process, defect sites initially develop on the pore walls in between the pores due to the local stress at the edges of each pore [21]. This allows for each individual dimple to act as a nucleation site for the solid state dewetting process once the metal film undergoes thermal treatment. In this approach, the local curvature gradient of the pores or dimples creates a chemical potential difference that drives the surface diffusion of the metal atoms. This results in a more ordered array of nanoparticles of controlled and predefined shape and location.

In this work, we present a detailed investigation of the effect of various experimental parameters on the ordering, shape, and size of gold and indium nanoparticle arrays. The nanoparticle arrays were formed on nano-dimpled aluminum substrates, produced using a two-step anodization growth procedure. They were then transferred to a substrate of choice using a polymer lift-off method. Anodized aluminum oxide (AAO) templates have been studied and used for decades [22]. Control of the spatial characteristics of the AAO template was achieved through a critical control of the anodization potential. The size of the metal nanoparticles was controlled by varying the anodization potential as well as the thickness of deposited metal during the solid state dewetting process.

## 2. Materials and Methods

High purity Al foils (Alfa Aesar, 99.9995% purity) were degreased using acetone, alcohol and de-ionized (DI) water in an ultrasonic bath. The foils were chemically polished at 85 °C using a chemical etch consisting of 15 parts 68% nitric acid and 85 parts 85% phosphoric acid. The foils were subsequently neutralized in 1 M sodium hydroxide for 20 min.

A two-step anodization process was used to form the dimpled surface [23,24,25,26,27,28]. To study the effect of anodization potential on pore ordering and pore size, the AAO membranes were grown for 30 min at different anodization potentials, ranging from 40 to 70 V. The polished Al foils were first anodized at room temperature using 1 M oxalic acid at a fixed potential, resulting in a thin porous oxide on the Al surface with randomly oriented pores. The Al foil with the thin oxide layer was then etched at 60 °C for 30 min in a mixture of 1.8% chromic acid (H${}_{2}$CrO${}_{4}$) and 6% phosphoric acid (H${}_{3}$PO${}_{4}$). Following this etch, the Al foils were anodized a second time for 2 h, under the same conditions as the first process. The resulting pores were then widened with 5% phosphoric acid at room temperature for 10 min. To create the nano-dimpled surface, the AAO membranes with the widened pores were etched in H${}_{2}$CrO${}_{4}$ + H${}_{3}$PO${}_{4}$ for 2 h.

To fabricate ordered Au nanoparticle arrays, two different thicknesses of the Au film (8 nm and 17 nm) were deposited on to the porous ‘nano-dimpled’ surface of the AAO membrane using a Turret Linde Four Position Auto Thermal Evaporator. The films were then induced to de-wet by annealing the films at 350 °C for 30 min using a Blue M Lab-Heat Box Type Muffle Furnace. To study the effect of nanoparticle size and ordering, the annealing times were varied from 30 min to a maximum of 2 h in ambient atmosphere. The de-wetted films were studied using a Hitachi Scanning Electron Microscope SU1510 and a Nanosurf Mobile S Atomic Force Microscope. The range of temperatures and times used in the de-wetting process helped identify the most optimum conditions for fabrication of an ordered array of spherical nanoparticles. The metal nanoparticles under study in this work include gold and indium.

**Figure 1:**
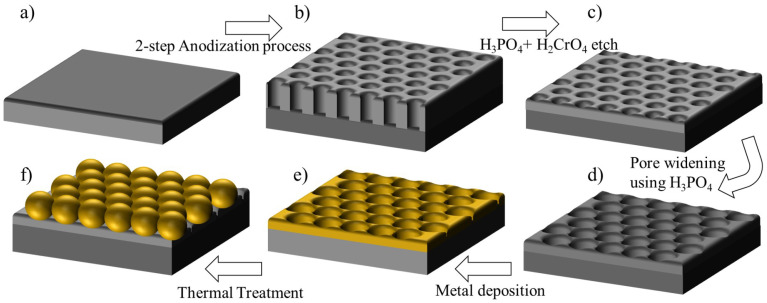
Schematic of the nano-dimpled Al surface (**a**–**c**) and synthesis of gold nanoparticle arrays (**e**,**f**). (**a**) Chemically polished Al foil; (**b**) Thick Al${}_{2}$O${}_{3}$ layer grown on Al surface, following a 2-step anodization process; (**c**) Chemical etch to thin the Al${}_{2}$O${}_{3}$ membrane to created a dimpled surface; (**d**) Dimpled Al${}_{2}$O${}_{3}$ layer with widened pores; (**e**) Thermally evaporated thin metal film conformally coating the dimpled surface; (**f**) Synthesis of an array of metal nanoparticles formed after thermal annealing.

The nanoparticle array formed in the dimples of the AAO membrane was transferred to a substrate of choice using polymethyl 2-methylcrylate (PMMA) (Figure 1). In this process, PMMA was spin-coated onto the nanoparticle array that was embedded in the dimpled membrane surface, allowing for a conformal encapsulation of the metal nano-particles within the PMMA film. Using a sharp cutting tool, the edges of the hardened region of the PMMA coated nanoparticle array was cut out of the Al foil while it was still submerged in DI water. This allows the DI water to penetrate between the PMMA film and the dimpled surface, causing the PMMA film to gently lift off the aluminum substrate and float to the surface of the water. This film is then gently scooped out of the DI water using the substrate of choice, allowing for the nanoparticles embedded within the PMMA film to come into direct contact with the transfer substrate. The PMMA film is subsequently dissolved by a few drops of di-chloromethane that was dropped onto the substrate surface. The residue was removed from the substrate surface using an acetone and DI water rinse.

## 3. Results

### 3.1. Engineering the Structural Properties of the Pores AAO Membrane

**Figure 2:**
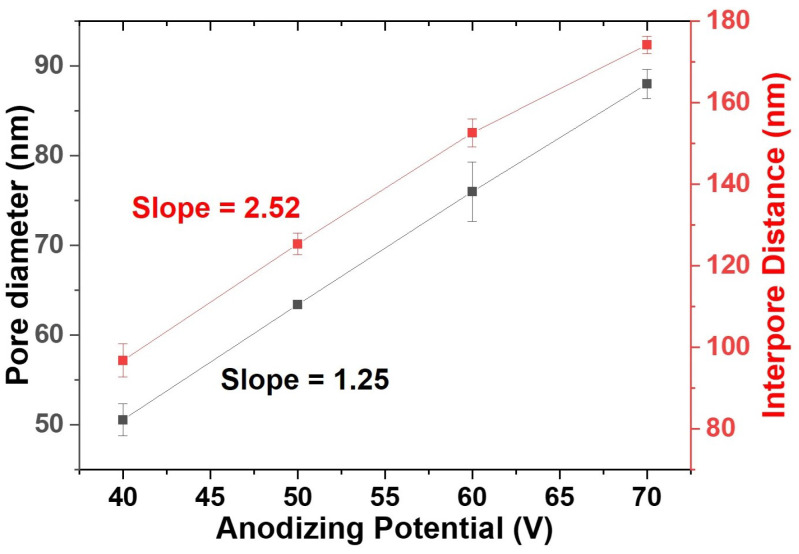
Plot representing the linear dependence of pore diameter and interpore distance on anodizing potential.

The structural parameters, mainly the diameter and interpore distance of the pores formed on the AAO membranes, were varied by varying the anodization potential for a 2 h growth. The results discussed below are for pores formed on the AAO membrane following the two anodization processes discussed in the previous section. It has been reported [29,30,31,32] that an increase in the anodizing potential increases the pore diameter and interpore distance according to the following equation:

(1) $$\begin{array}{c} D_{P}=\varepsilon_{P}\times U=1.29\times U\\ D_{int}=\varepsilon_{int}\times U=2.5\times U \end{array}$$

where $D_{P}$ and $D_{int}$ are the pore diameter and distance between pores, $\varepsilon_{P}$ and $\varepsilon_{int}$ are the proportionality constants for pore diameter and distance between pores, and *U* is the anodization potential. The linear correlation between $D_{P}$ and $D_{int}$ was tested for anodization potentials varying from 40 V to 70 V in steps of 10 V. Figure 2 is the plot showing the variation of $D_{P}$ and $D_{int}$ with anodization potential. The slopes of the two curves agree with the proportionality constants $\varepsilon_{P}$ and $\varepsilon_{int}$ in Equation (1).

**Figure 3:**
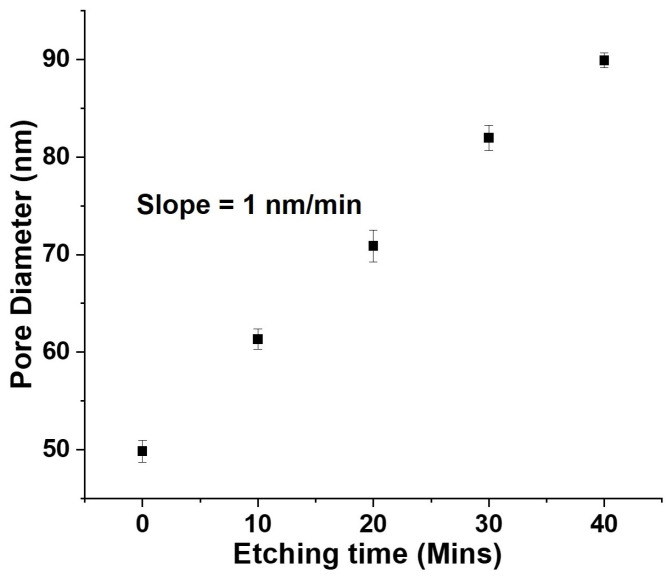
Correlation between the pore diameter and etching time, using data acquired from SEM imaging; slope representing an approximate etch rate of 1.0 nm/min.

Following anodization, the pores formed in the AAO membranes were widened by treating the sample with H${}_{3}$PO${}_{4}$. The pore-widening step by an isotropic chemical etching process will etch all of the walls within the pores at the same rate [33,34,35], thereby enabling control over the pore diameter, pore depth and the angle of curvature. The dependence of the pore diameter on the etching time is shown in Figure 3. The slope, representing an etching rate of approximately 1.0 nm/min, is in close agreement with previously reported etching rate [29]. The pore-widening step is critical to the formation of the nanoparticle array; details of which will be covered in the next section.

**Figure 4:**
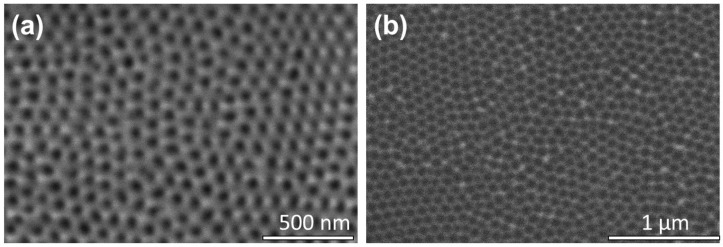
SEM image of: (**a**) the bare AAO template following the second 2 h anodization and pore widening for 10 min; (**b**) the nano-dimpled membrane after the second chemical etch that reduced the height of the pores.

The fabricated porous AAO membrane, following the second anodization, was comprised of pores that were tens of microns long and tens of nm wide. The pore widening step (results shown in Figure 3) was performed on this microns high membrane to produce pores of different diameters. This membrane is shown in Figure 4a. This was followed by the second chemical etch to modify the aspect ratio of the pores by reducing the pore heights to tens of nm. The pores in the AAO membrane is thereafter referred to as dimples, with the SEM image shown in Figure 4b.

### 3.2. Fabrication of Ordered Array of Metal Nanoparticles

The results presented in this section are for dimpled AAO membranes fabricated at 40 V. The local geometry of the dimples were altered by the H${}_{3}$PO${}_{4}$ etch for 10 min, resulting in deeper dimples and thinner walls. The fabrication process and the dewetting theory was validated for two different arrays of nanoparticles—gold (Au), and indium (In)—that were obtained by thermal evaporation of the metal onto the dimpled surface. On ideal surfaces, dewetting is initiated at inhomogeneities such as defects, film edges or grain boundaries. It can also be initiated at specific sites by using a patterned substrate, where the process is driven by a defined surface topography, like the dimples formed in the AAO membrane [20]. Each individual dimple on the membrane provides a short length scale artificial curvature modulation, allowing it to act as a nucleation site for the solid state dewetting process. The dewetting process in this case occurs at a lower temperature compared to flat surfaces and is driven by a curvature-induced diffusion mechanism [36], where the ridges around the dimples act as diffusion barriers, trapping the metal into the valleys of the dimple. The characteristic dewetting temperature for thin metal films deposited on patterned surfaces is reported to be the Hüttig temperature [36,37], which is the temperature at which the metal atoms at defect sites become mobile. The melting point of bulk Au is 1064 °C, while the Hüttig temperature is about 319 °C [38]. In general, the Hüttig temperature is reported to be about 30% of the melting point of the metal [37,39,40].

The local curvature of the dimples is related to the surface energy of the film, and the local chemical potential through the Gibbs-Thomson relationship:

(2) Δμ=κγΩ

where Δμ describes the local chemical potential, κ is the local curvature, γ is the surface energy, and Ω is the atomic volume [41]. Repetto et al. [42] and Giermann et al. [19,20] have done detailed studies on the dewetting process on patterned substrate surfaces. Their work discusses the role of surface tension in creating instabilities in the film to control the solid-state de-wetting process, which results in an ordered array of near-uniform size nanoparticles. For films above a certain thickness, the surface tension in the film drives the curvature-induced diffusion process, causing a redistribution of the metal film volume in the local area of the dimple. The driving force behind this volume redistribution is the local flux of surface metal atoms from the positive curvature regions on the membrane walls to the negative curvature regions in the valleys of the dimples. This flux is caused by the local curvature gradient that is formed when the evaporated film conforms to match the geometry of the patterned surface. If the surface energy is isotropic, this surface flux is given by [43]

(3) $$\begin{array}{c} \vec{J}=-\left(\frac{D_{s}\gamma_{fa}N_{s}\Omega }{k_{B}T}\right)\nabla_{s}\kappa \end{array}$$

where $\vec{J}$ is the surface flux of metal atoms, $D_{s}$ is the surface diffusivity, $\gamma_{fa}$ is the surface energy, $N_{s}$ is the number of surface atoms per area, Ω is the atomic volume, $k_{B}$ is the Boltzmann’s constant, *T* is the temperature, and $\nabla_{s}\kappa$ is the local gradient of surface curvature. The gradient $\nabla_{s}\kappa$ can be influenced both by the geometry of the underlying patterned surface and by the thickness of metal that is deposited onto the surface.

**Figure 5:**
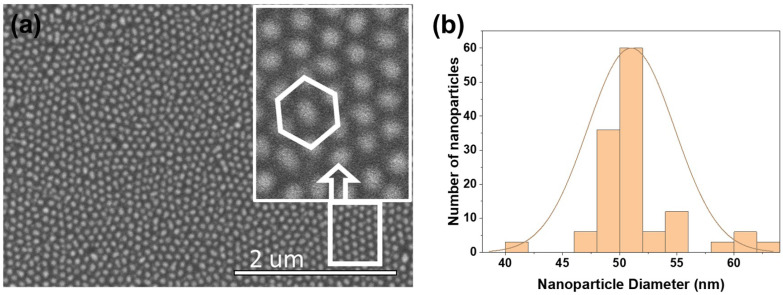
(**a**) SEM image of the AAO membrane surface following dewetting using 8 nm of gold. The inset is an enlarged view of the array showing hexagonal close-packed ordering. (**b**) Distribution of gold nanoparticle size taken over a 1.0 μm${}^{2}$ area.

In the first part of this study, the geometric features of the AAO membrane, like the dimple diameter and the wall thickness, are held fixed. The kinetics of the dewetting process were analyzed by studying the morphology of an Au nanoparticle array for two different thickness of the deposited gold: 8 nm and 17 nm, followed by annealing at 350 °C in ambient conditions for 30 min. Figure 5a is an SEM image of the nanoparticle array morphology for an 8 nm thick Au film. An ordered structure (hexagonal close-packed ordering) with large areal coverage and a single nanoparticle occupying each dimple was observed, as shown in the inset of Figure 5a. The average nanoparticle size was estimated to be about 50 nm, with an inter-particle spacing of about 30 nm. Using the Quartz PCI analysis software on the Hitachi SEM, the nanoparticle size distribution over a 1.0 μm${}^{2}$ area was estimated and the statistical analysis of the size distribution is shown in Figure 5b. The imperfections in the Al foil were transferred to the nanoparticle array pattern, which shows individual domains and some voids. This is attributed to the fact that the Al foils were not electro-polished, but were only chemically etched prior to AAO membrane formation. A more uniform pattern would evolve with electro-polished, annealed foils of Al.

**Figure 6:**
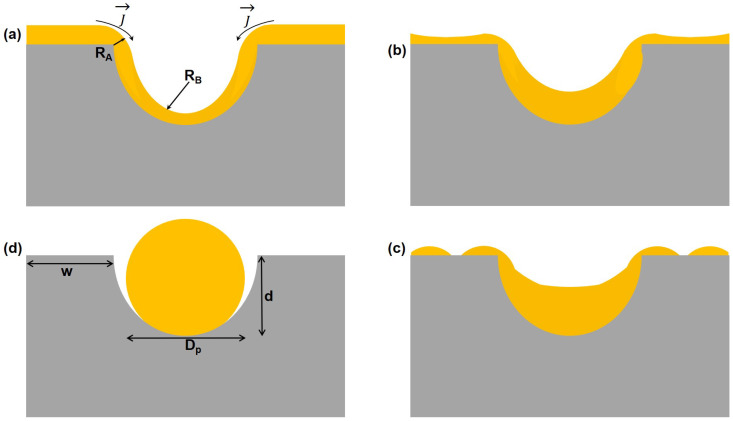
Schematic illustration of curvature-driven evolution of the metal film. (**a**) A conformal film coating the dimpled surface. The curvature at the edge and at the bottom of the dimple is 1/$R_{A}$ and 1/$R_{B}$, respectively; (**b**) The surface diffusion from A to B (shown by arrows in (**a**)) is a consequence of the film attempting to minimize the local curvatures as the metal begins to diffuse from the edges into the dimples; (**c**) Breaking up of the metal film on the walls, caused by the flux of atoms into the dimples. (**d**) For thin films and narrow walls, a single particle forms in each dimple.

To explain the ordered array formation, consider the schematic of Figure 6. At the edge of the dimple, the conformal film has a curvature $\kappa_{A}$ = 1/$R_{A}$, while at the bottom of the dimple the curvature is $\kappa_{B}$ = 1/$R_{B}$, as shown in Figure 6a. The film evolves to minimize these local curvatures by surface diffusion from A to B, resulting in a mass flux from the top corners of the surface of the film to the surface of the film above the tip of the pit. One consequence of this flux of atoms is the thinning of the film on the walls as the flux of atoms progressively diffuse into the valleys of the dimples. Once the film on the walls becomes thin enough, Van der Waals forces begin to overcome the action of surface tension that is holding the film together. This causes the film to split, exposing a portion of the walls and forming a hole in the film [42], as depicted in Figure 6b. The metal remaining on the walls then diffuses away from this hole and into the surrounding dimples, as shown in Figure 6c. The ridges of the dimples act as diffusion barriers trapping the metal inside, resulting in spherical nanoparticles buried in individual dimples, as shown in Figure 6d.

**Figure 7:**
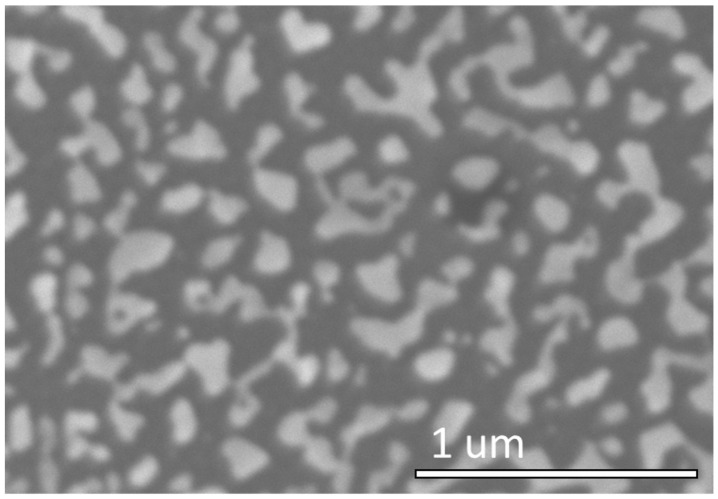
SEM image and schematic for results using 17 nm of deposited gold.

For thicker Au films (17 nm), the random formation of particles and islands, seen in the SEM image of Figure 7, indicates that the de-wetting process did not follow the surface topography. With a thick film, the local curvature gradient is too small to induce the curvature driven diffusion that is observed for thinner films. The thicker film therefore produced a de-wetted film, with formation of large islands with a broad size distribution, a characteristic typically observed in de-wetted films on flat surfaces.

The study above indicates that there exists a strong correlation between the film thickness, the dewetting process, and the morphology and ordering of the nanoparticle array. For each pore diameter, there is a critical thickness of the deposited film that yields uniform spherical nanoparticles, with single nanoparticle occupying each pore. This allows for an ordered array of uniform sized nanoparticles. A semi-quantitative analysis was used to demarcate sample geometries for which the metal film de-wets into the dimples from those which have material remaining on the walls and in the dimples. In the model developed by Giermann et al. [20], the authors considered an array of pits with inverted pyramidal shapes. They studied the nanoparticle morphology evolution during the de-wetting process as a function of the film thickness and the wall-width-to-period ratio. This model may be used to explain an optimal thickness value when using a patterned surface for dewetting. If the volume of the evaporated film is equal to the volume of the dimples, all of the metal will de-wet into the dimples resulting in a single particle occupying the dimple during the annealing treatment. Considering the dimples to have an ellipsoidal geometry, the volume of the dimple is defined by the equation:

(4) $$\begin{array}{c} V_{dimple}=\frac{1}{6}\pi D_{P}^{2}d \end{array}$$

where *d* is the depth of the dimple and $D_{p}$ is the dimple diameter. The volume of the conformal metal film is defined by the equation:

(5) $$\begin{array}{c} V_{film}={\left(D_{P}+w\right)}^{2}h \end{array}$$

where *w* is the thickness of the pore wall and *h* is the evaporated metal thickness. In order to study nanoparticle morphology evolution as a function of the film thickness and dimple geometry, the condition in which the volume of the film becomes equal to the dimple volume, for a complete dewetting of the dimples needs to be determined, where

(6) $$\begin{array}{c} R_{dh}=\frac{6}{\pi }{\left(1+R_{wD}\right)}^{2} \end{array}$$

In Equation (6), $R_{dh}=\frac{d}{h}$ is the ratio of the depth of the dimple to the film thickness. $R_{wD}=\frac{w}{D_{p}}$ represents the ratio of the wall thickness to the diameter of the dimple.

**Figure 8:**
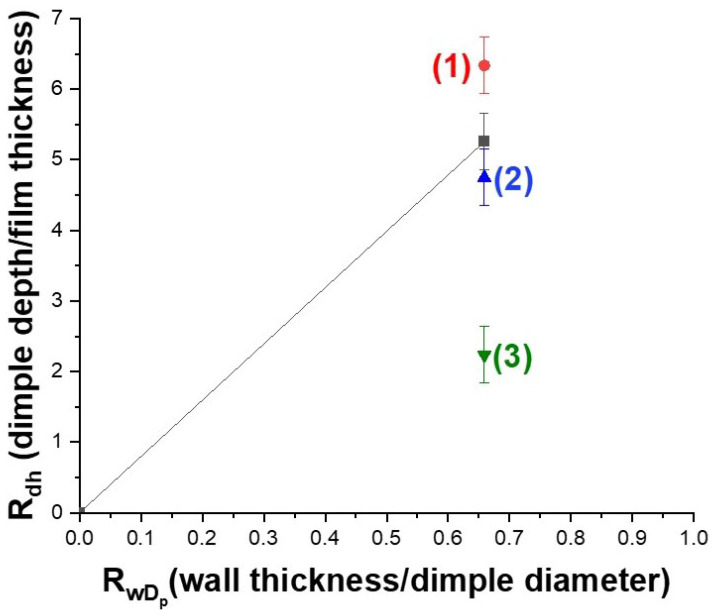
Dependence of nanoparticle morphology on metal film volume and dimple geometry. The trend line represents the condition in which the metal film volume is equal to the dimple volume. Three points labelled (1), (2) and (3) represent the location of $R_{dh}$ with respect to the trend line for films of thickness 6 nm, 8 nm and 17 nm, respectively.

Figure 8 describes the various nanoparticle morphologies and ordering observed as a function of the deposited film thickness. The solid line in Figure 8 represents the condition in which the volume of the film is equal to the dimple volume. An optimal film thickness of about 7 nm was obtained by applying the equality condition to a dimpled surface fabricated at 40 V. The height of the dimple for this case was measured by AFM and determined to be 38 nm. The significance of the volume equality condition is that it clearly demarcates sample geometries for which the metal film completely de-wets into the dimples (providing an ordered array of spherical nanoparticles) from those structures that have metal covering the walls, thereby disrupting the nanoparticle ordering. As seen in Figure 8, for a fixed $R_{wD}$ ratio of ≈ 0.7, three different morphologies were observed for different film thicknesses. Label (1) was assigned to the case where a film thickness of 6 nm resulted in a value of $R_{dh}$ that is above the solid trend line predicted by the model. The optimum condition was met for a film thickness of 8 nm (Label (2)). The model was tested for very thick films of about 17 nm (Label (3)) in Figure 8. This point clearly represents the case where the film thickness is significantly shifted from the theoretically predicted trend line, representing a cluster of metallic islands with no ordering.

**Figure 9:**
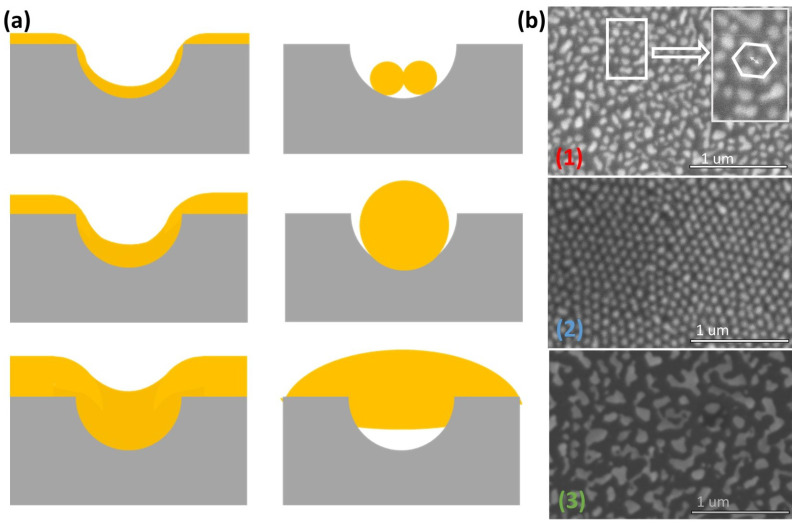
Morphology evolution following de-wetting process: (**a**) Schematic of conformal films of varying thickness on the dimpled surface; (**b**) Corresponding SEM images of nanoparticle arrays for films of varying thicknesses.

The de-wetting theory was applied to analyze the three geometries (labelled 1–3) in Figure 9, in which single particles form in the dimples with no material on the walls from those in which some material is on the walls resulting in island formation. As shown in Figure 9a, for very thin films of Au (Case 1), multiple particles form in the dimples, verified by the corresponding SEM image in Figure 9b. For the optimal film thickness of 8 nm (Case 2), single particles from in the dimple resulting in an ordered nanoparticle array. For Case 3, a fully de-wetted thick film (17 nm) results in large islands with a broad size distribution and no evidence of the dimpled array.

**Figure 10:**
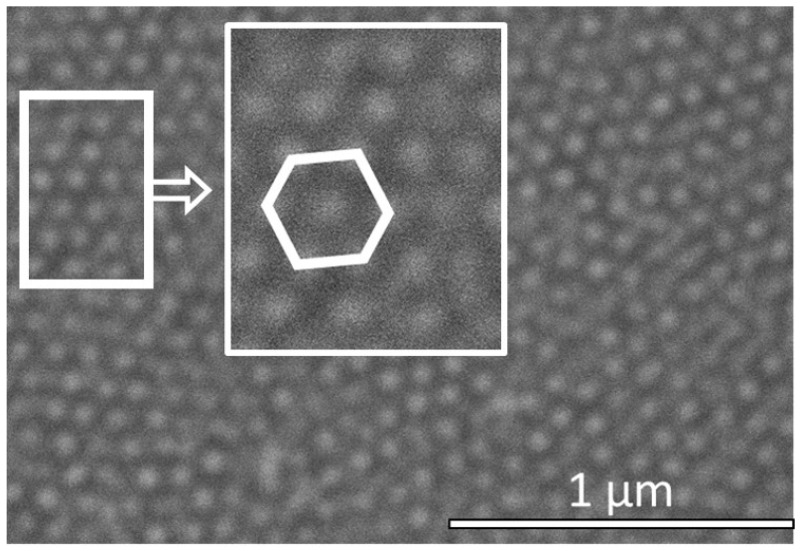
SEM image of indium nanoparticle array formed after evaporating 8 nm thick In film onto a dimpled surface fabricated at 40 V.

The previous discussion was for Au films. To validate the dewetting theory and the model discussed above, an 8 nm indium (In) film was deposited on the dimpled membrane using an anodization potential of 40 V. Figure 10 is an SEM image of the ordered array of In nanoparticles. Considering the low melting point of In, the annealing temperature for these films was 60 °C. The ordered array is comprised of 50 nm In nanoparticles, with a single particle occupying each dimple and no metal on the dimple walls. These results clearly validate the model.

## 4. Conclusions

In this work, the conditions required for fabricating ordered metal nanoparticle films on topographically patterned dimpled surfaces were analyzed. It was found that the thickness of the evaporated metal film is related to the curvature of these films on the dimpled surface. This curvature modulation controls the initiation of the solid-state dewetting process that results in uniform sized nanoparticles in the patterned array of dimples. Several different morphologies formed during the film de-wetting process. A simple model that correlates the volume of the evaporated film to the dimple volume was used to demarcate film thicknesses and morphologies that deviated from the trend line. For the given geometry (ellipsoidal) of the dimples, an optimum thickness of 7 nm was estimated to yield an ordered array of uniformly sized metal nanoparticles. Thick films resulted in formation of randomly distributed islands on top of the dimpled topography. On the other hand thin films resulted in the formation of multiple nanoparticles inside the pits. For optimum conditions, particles formed inside the pits resulted in an ordered array. The de-wetting process, based on curvature-driven surface smoothing and the model to optimize film thickness for a given pore geometry, was validated for Au and In films. This work provides a semi-quantitative analysis of the conditions for de-wetting to form uniform periodic arrays of metal nanoparticles.
